# The Role of Full-Thickness Skin Grafts in Patient’s Rehabilitation after Maxillectomy and Midface Defects

**DOI:** 10.3390/jcm11133608

**Published:** 2022-06-22

**Authors:** Iwona Niedzielska, Łukasz Obszyński, Michał Bąk, Damian Niedzielski

**Affiliations:** Department of Craniomaxillofacial Surgery, Medical University of Silesia, 40-027 Katowice, Poland; iniedzielska@sum.edu.pl (I.N.); niedzielskidamian@gmail.com (D.N.)

**Keywords:** full-thickness skin, maxillectomy, midface defects

## Abstract

(1) Background: Nowadays, microvascular grafts are the gold standard in the reconstruction of midface defects after maxillectomy, however, not all patients may qualify for this type of surgery. The purpose of present study is to evaluate the benefits of alternative reconstruction methods such as full-thickness skin grafts for these conditions. (2) Methods: The research group consisted of 37 patients who underwent maxillectomy due to cancer of the mid-face and had full-thickness skin graft reconstruction. The study covered the period from 2011 to 2020. (3) Results: Based on the clinical examination and the subjective assessment of patients, a positive effect of the use of free skin grafts on their convalescence and rehabilitation was found. In particular, they contributed to the reduction in postoperative pain and pain associated with prosthetic stages (VAS Scale). (4) Conclusion: Full-thickness skin grafts in combination with individual prosthetic restorations are a good alternative to rehabilitation in patients who do not qualify for microsurgical treatment.

## 1. Introduction

Neoplastic disease of the mid-face area constitutes 10% of head and neck neoplasms. Malignant lesions of this region are 7 times more frequent than benign lesions. Their morbidity is estimated at about 0.7 per 100,000 inhabitants. Due to the very frequent expansion towards the maxillary and ethmoidal sinus, clinical symptoms present at a later stage and their course is strictly dependent on the location, size, and direction of tumor infiltration. The most common symptoms reported by patients are pain in the mid-face area, pathological exudate, nasal obstruction, facial asymmetry, sensory disturbances in the maxillary nerve and visual disturbances [1,2].

The diagnosis of neoplasms of the mid-face is based on clinical examination, rhinoscopy, endoscopy, computed tomography (CT), magnetic resonance imaging (MRI) and histopathological examination. Due to the non-specific course of the disease and diagnostic difficulties, most patients are diagnosed at stage T3 and T4 [3].

The most frequently chosen treatment modality is surgery, often supported by radiotherapy, less often by chemotherapy. The main goal of surgical treatment is a complete tumor removal with negative resection margins [4,5,6].

Oro-nasal communication, loss of teeth and bony structures of the middle part of the face, facial asymmetry are the main consequences of surgical procedures within the maxillofacial ethmoid massif. Consequently, activities such as talking, chewing, and swallowing are severely impaired [7].

Nowadays, the reconstruction of post-operative extensive tissue defects is most challenging. There are numerous techniques of surgical reconstruction such as microvascular reconstructions, individual implants, and prosthetic combined appliances with zygomatic implants.

Tissue transplantation on microvascular free flaps is recognized as the best standard of surgical treatment [8,9]. Due to the length and complexity of the procedure and the high risk of failure of this method, not every patient can be qualified for this type of treatment [10]. Elderly patients with general diseases, which make it impossible to carry out long-term surgery, require the use of alternative surgical techniques enabling a quick return to social life and effective post-operative rehabilitation [11,12,13,14]. 

The aim of the study is to assess the usefulness of full-thickness skin grafts used to treat the mid-face defects of the jaw in oncological patients. 

## 2. Materials and Methods

The study included 52 patients surgically treated for jaw tumors, from whom a group was collected, in which free skin graft from the groin area was used. The study took place at the Department of Cranio-Maxillofacial Surgery in Katowice from January 2011 to June 2021. The group consisted of adult patients without predilection for age and gender. The exclusion criteria allowed patients with health disorders and those using stimulants (alcohol, cigarettes). The study group consisted of 37 patients with a free skin graft from the groin area, while the control group consisted of 15 patients who did not consent or were not qualified for free skin graft because of local contraindications (dermatological skin diseases of groin area, urinary tract infections UTI, perianal diseases, such as anal fissures and hemorrhoids). All of the patients were supplied with a palatal plate in the operative period.

**Figure 1:**
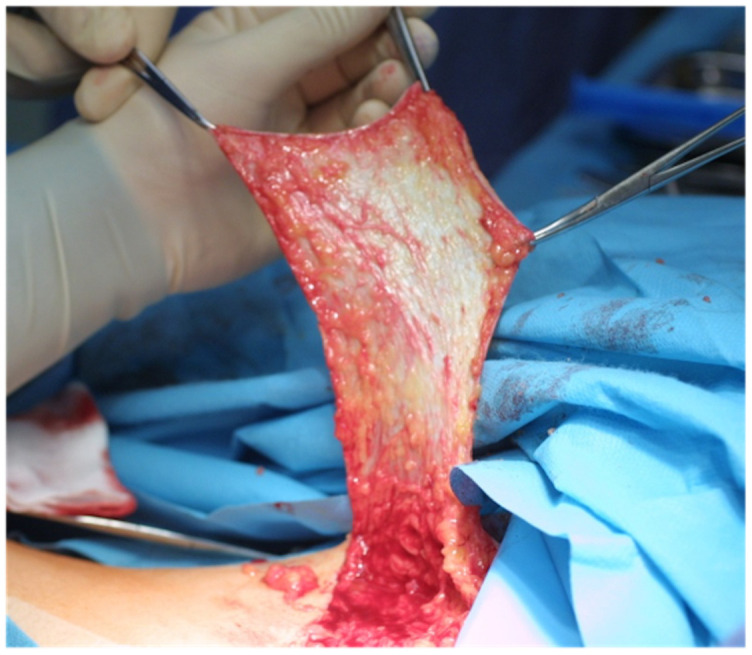
Free full-thickness skin graft harvesting from the groin area.

**Figure 2:**
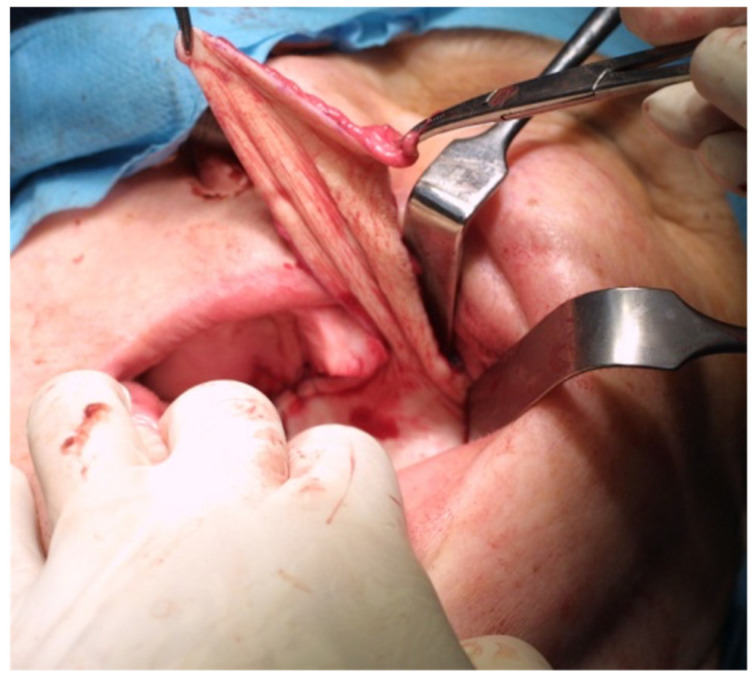
Full-thickness skin graft sewing.

**Figure 3:**
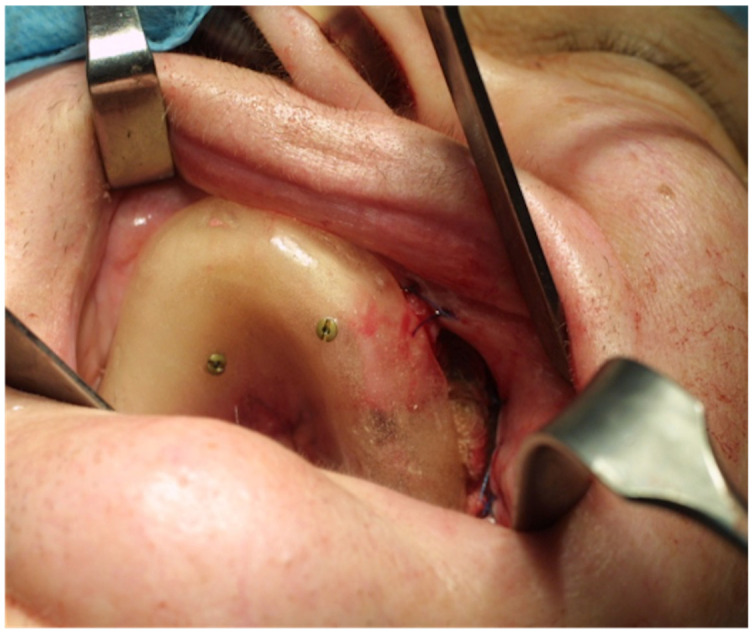
Full-thickness skin graft stabilized with a gauze dressing and a previously made palatal plate attached with a bicortical titanium screws.

Patients qualified for surgery had to undergo a complete diagnostic test panel to get prepared for the procedure. The surgical procedure in study group consisted of complete tumor removal with negative resection margins (the radicality of the resection was assessed by intraoperative histopathological examination), a possible neck dissection and subsequent replenishment of the post-resection area with free full-thickness skin graft from the groin area (Figure 1). The size of the graft corresponded to the size of the recipient site, considering the excess tissue resulting from the shape of the graft (lenticular) which enabled better adaptation of tissues around the donor site. The graft was cleared of adipose tissue and perforated with a needle to increase its dimensions, flexibility and allow drainage, preventing the formation of a hematoma after its introduction into the recipient site (Figure 2). After being sewn into the post-resection area, the free skin graft was stabilized with a gauze dressing with iodoform and a previously made palatal plate attached with a bicortical titanium screw to the preserved fragment of the hard palate or maxilla alveolar process (Figure 3) [15]. After the mobilization of the surrounding tissues, the donor site was sutured in layers. In the control group, the post-resection defect was supplied with gauze dressing with iodoform, and a palatal plate stabilized with a screw.

After surgery, patients were fed with a feeding tube or with percutaneous endoscopic gastrostomy (PEG). During hospitalization, on the 10th day the palatal plate and gauze dressing were removed, the healing was assessed, any necrotic tissues were removed, and an impression was taken under the obturator. The rate of flap healing in the study group was classified into 2 groups: correct, partial; abnormal, complicated by inflammation. After the post-resection area had fully healed, the patients were qualified for adjuvant radiotherapy: 29 (78%) in study and 13 (86%) in control group. Final prosthetic restoration was performed after full recovery (retention of appliances was obtained by using tooth clamps, dental, zygomatic or titanium individual implants).

The evaluation of the patient’s convalescence and the effectiveness of rehabilitation in the research and the control group was evaluated on the basis of the patient’s subjective assessment in a medical records and questionnaire survey carried out by telephone. In the postoperative period, patients were provided with painkillers if needed *(paracetamolum, deksketoprofenum)* and were asked not to take them on the day of the control. This study included the assessment of pain symptoms (VAS scale) in 10th day after the procedure, followed by taking the impression under the palatal plate (up to 28 days after the procedure) and the subjective assessment of the ability to speak and eat in the post-operative period [16].

This study followed the Declaration of Helsinki on medical protocol and ethics and was approved by the Bioethical Committee of Medical University of Silesia (ethics approval number KNW/0022/KB1/146/12).

## 3. Results

The study group included 19 men and 18 women (M:F = 1.06), with an average age of 61.65 years (the youngest patient was 37 years old, the oldest was 81 years old). In the studied cases, the tumors were at the stage of T1-T4 (average tumor size 4 cm^2^), and the most common histopathological diagnosis was Squamous Cell Carcinoma (SCC) (51%). (Table 1) The mean stage of the lymph node criterion was N0 (43.2%), and of the organ metastases M0 (84.0%). In most cases (48%), the surgical procedure consisted of a subtotal resection of the maxilla with selective neck dissection followed by subsequent replenishment of the post-resection area with free full-thickness skin graft. The midface defects were classified in Brown’s vertical classification as I and II; in horizontal as a, b, c, d. The average size of the harvested graft was 7 cm^2^. 

Among patients in the control group, there were 9 men and 6 women (M:F = 1.5) with an average age of 67.6 years. The most common histopathological diagnosis was also Carcinoma planoepitheliale (80%) in the T1–T4 stage. The midface defects were classified in vertical and horizontal Browns’ classification similarly as in study group (I, II, a, b, c, d).

Full hospitalization lasted about 11 days (10.94). On average, on the 10th day (9.94) the palatal plate was removed, and the first assessment of the local condition was made. Further controls of the healing took place in ambulatory care center. In study group, proper healing of the graft was achieved in 30 (81.1%) cases, while in 7 (18.9%) there was an inflammatory complication. The average time of the full healing of the flap was 29 days (28.51) (Table 2). In the control group, a longer healing process was observed (35.1 days) with more frequent inflammatory healing complications (ulcers, purulent inflammations—28%). The result of the final histopathological examination confirmed the radicality of the procedure.

The next stage of the study was the assessment of patient’s rehabilitation based on medical records and telephone surveys. Based on the obtained responses in study group, pain symptoms on the 10th day after the procedure were assessed on the VAS scale at an average of 5.48 points; at the time of taking, the impression for the postoperative palatal plate 6.65 and 4 weeks after the procedure at 2.7. In the control group it was, respectively, 5.2, 7.6 and 4.6. The quality of speaking and eating, where 1-no ability to speak/eat, 10- no difficulty in speaking/ eating, was assessed, respectively, in study group at 6.8/7.9 and in control group at 5.8/6.0. The main complaint reported by patients (47%) was insufficient stability of the used obturator (Table 3).

## 4. Discussion

Reconstruction of post-resection midface defects is an integral part of the surgical treatment of patients with head and neck neoplasms. The development of technology has contributed to the creation of numerous methods of reconstruction aimed at obtaining the optimal esthetic effects, postoperative comfort, but also the elimination of complications. [17,18].

Nowadays, microvascular grafts are considered the “gold standard” of treatment. [19] However, in many cases this solution is not feasible due to the patient’s general health condition and the degree of advancement of the disease [20]. This fact was an incentive for the authors to seek solutions that would improve the treatment methods used so far. The results of the available studies clearly prove the effectiveness of the use of obturators in patients who have undergone maxillectomy [21,22]. The main disadvantage of this solution is severe pain associated with the initial period of use. Thanks to the use of full-thickness skin grafts, this problem is eliminated, which was determined based on the conducted study.

Due to the large amount of tissue in this place and the invisibility of the scars, the use of the groin area as a donor site seems to be optimal. The literature describes the use of free skin grafts after resection of the maxilla: subtotal maxillectomy, total maxillectomy. In each of these procedures, a skin graft is designed to cover and protect the exposed bone surface in the tumor resection bed, facilitating wound healing [23].

When controlling the healing of free skin grafts, it was not expected that it could heal completely, especially in its central part due to the lack of substrate but healing of the adjacent soft tissues was expected. In this situation, the free skin graft additionally performed the function of a dressing, which, together with the palatal plate, ensured a tight separation of the oral cavity from the nasal cavity. This resulted in optimal healing conditions for the tissues of the resection bed and a significant reduction in the pain. The most important thing is the fact that the surveyed patients improved their comfort and reduced postoperative pain, especially at the stage of making impressions in order to perform prosthetic restorations.

During the further use of obturators, patients positively assessed their pronunciation and communication with the society. They also reported no significant food intake problems. The most frequent complaint was the poor stability of prosthetic restorations.

Davidson and Sherris seek insufficient stability of prostheses in excess tissues due to healed free skin graft; however, due to the retention mechanism of the anchoring of restorations, this conclusion was not confirmed in the analysis conducted by the authors [24].

According to Brown and Rogers, obturators are a useful reconstruction method in all types of maxillectomy classification [25]. Relating to poor stability of this appliances, it seems to be very essential and challenging to optimize and improve quality of this restorations, which was the primary aim of the following study.

One of the possibilities to increase the stability of the obturator is implants placement around the surrounding healthy bone tissue. This enables better anchorage of the obturator and prevents micromovements and traumatization of surrounding tissues, reducing the risk of recurrence of the neoplastic process [26].

Heckett, El-Wazanii in their meta-analysis of 437 publications of the usage of zygomatic implants in patients with midface defects after maxillectomy unequivocally assess the validity of their use—especially in a one-stage protocol, due to the high probability of subsequent radiotherapy of the area, which significantly reduces the chances of osteointegration the implant [27].

Based on the study, it can be concluded that the use of free skin flaps with an obturator is a particularly good method of treatment in patients who do not qualify for more advanced reconstructive methods, such as microvascular reconstructions. They allow for a reduction in pain in the perioperative period and reduce the risk of recurrence of the disease. The obturators used function as a temporary restoration, while the final restorations require additional anchoring in the form of implants.

Another important advantage of the presented methodology, using obturators, is the simplicity of the procedure presented by Brandao and Migliorati, which may be an excellent alternative to microvascular reconstructions in the time of the COVID-19 pandemic and the related logistic and personnel constraints [28].

## 5. Conclusions

Full-thickness skin grafts ensure optimal healing conditions and reduce postoperative pain after maxilla resection surgeries. In combination with individual prosthetic restorations, they are a suitable alternative for rehabilitation in patients who do not qualify for microsurgical treatment.

## Figures and Tables

**Table 1:** Structure of groups.

		Study Group		Control Group	
		Number of Patients	% Patients	Number of Patients	% Patients
Sex	Female	18	49	6	40
	Male	19	51	9	60
Age	30–50	10	27	1	6.7
	50–70	17	46	6	40
	>70	10	27	8	53.3
Type of Neoplasma	Carcinoma planoepitheliale	19	51	12	80
	Adenoid cystic carcinoma	4	11	1	6.7
	Mucoepidermoid carcinoma	3	8	0	0
	Others	11	30	2	13.3
Tumor Size	<2 cm	10	27	5	33.3
	2–4 cm	22	59	7	46.7
	>4 cm	5	14	3	20

**Table 2:** Assessment of flap’s healing.

		Study Group		Control Group	
		Number of Patients	% Patients	Number of Patients	% Patients
Time of Full-Healing	<14	1	3	0	0
	14–28	21	57	7	46.7
	>28	15	40	8	53.3
Healing	correct, partial	30	81	-	-
	abnormal, complicated by inflammation	7	19	-	-

**Table 3:** Assessment of patient’s rehabilitation.

		Study Group		Control Group	
		Number of Patients	% Patients	Number of Patients	% Patients
Pain in the 10th day after surgery (VAS)	<5	9	24.3	3	20.0
	5–8	28	75.7	12	80.0
	>8	0	0	0	0
Pain when taking the impression (VAS)	<5	5	13.5	0	0
	5–8	25	67.6	11	73.3
	>8	7	18.9	4	26.7
Pain after 4 weeks after surgery (VAS)	<5	34	91.9	6	40.0
	5–8	3	8.1	9	60.0
	>8	0	0	0	0
Subjective evaluation of the quality of speaking	1–4 (limited)	2	5.4	4	26.7
	5–7 (suffiecient)	23	62.1	9	60.0
	8–10 (correct)	12	32.4	2	13.3
Subjective evaluation of quality eating	1–4 (limited)	0	0	1	6.7
	5–7 (suffiecient)	11	29.7	11	73.7
	8–10 (correct)	26	70.3	3	20.0

## Data Availability

The data presented in this study are available on request from the corresponding authors. Publicly data sharing is not applicable to this article due to privacy policy.
